# Combining species distribution models and big datasets may provide finer assessments of snakebite impacts

**DOI:** 10.1371/journal.pntd.0012161

**Published:** 2024-05-20

**Authors:** Mohammad Abdul Wahed Chowdhury, Johannes Müller, Aniruddha Ghose, Robed Amin, Abdullah Abu Sayeed, Ulrich Kuch, Mohammad Abul Faiz

**Affiliations:** 1 Department of Zoology, University of Chittagong, Chattogram, Bangladesh; 2 Museum für Naturkunde, Leibniz-Institut für Evolutions- und Biodiversitätsforschung, Berlin, Germany; 3 Institut für Biologie, Humboldt-Universität zu Berlin, Berlin, Germany; 4 Venom Research Centre, Department of Medicine, Chittagong Medical College, Chattogram, Bangladesh; 5 Department of Medicine, Chittagong Medical College, Chattogram, Bangladesh; 6 Directorate General of Health Services, Ministry of Health and Family Welfare, Dhaka, Bangladesh; 7 Institute of Occupational Medicine, Social Medicine and Environmental Medicine, Goethe University, Frankfurt am Main, Germany; 8 Dev Care Foundation, Chattogram, Bangladesh; University of Massachusetts Amherst, UNITED STATES

## Abstract

**Background:**

Snakebite is a major poverty-related neglected tropical disease. An integrated scientific approach is needed to understand the dynamics of this important health issue. Our objective was to estimate snakebite occurrence in a tropical area by using a blend of ecological modelling and robust statistical analysis.

**Methods:**

The present study used climatic, environmental, and human population density data to determine the area with snakebite occurrence-probability for the first time in Bangladesh. We also analysed a large, 16-year dataset of hospitalized snakebite cases to reveal the epidemiology of snakebite in the south-eastern zone of the country.

**Findings:**

Our results show that cobra bite is the most commonly occurring venomous snakebite in humans (around ~12% of the total yearly snakebite records), and men are more frequently bitten than women (2/3 of human victims are men). Most bites occur during the rainy season for cobra and green pit viper, while krait bites are not restricted to any particular season. As snakebite incidents are closely related to climate conditions, we can model snakebite risk using temperature and precipitation variables. Whereas there is a lack of snakebite reports from several parts of the study area in official records, our models predict that the entire study area is favourable for snakebite incidents. Based on the combined evidence we estimate that about 200,000 snakebite events occur every year in the south-eastern part of Bangladesh alone. Considering future global climate change, our model projections show that snakebite incidence in Bangladesh might not significantly decrease in the future (- 2070-); however, the distribution of probabilities might change, with a predicted increase of snakebite incidence in the hilly areas of the country.

**Conclusions:**

Using climatic data to predict snakebite incidence in Bangladesh allowed us to provide estimations of the total annual number of snakebite cases in the study area. As in most countries, the scarcity of accurate epidemiological data in official records might have masked the real magnitude of this problem. Our analysis suggests that the problem of snakebite envenoming in Bangladesh might be worse than currently perceived. A long-term sustainable snakebite program plan should be designed and institutionalized, considering climatic, geographical and human demographic variables, to obtain better data and facilitate the implementation of accurate snakebite management programs for this country.

## Author summary

Snakebite envenoming is an important health problem in Bangladesh. A community-based survey conducted in 2009 indicated that around 700,000 snakebites/year may occur in this country. However, until now, there is no study estimating the incidence of snakebite in Bangladesh from the aspect of ecology. Considering the climatic, environmental, and human population variables, a large, 16-year clinico-epidemiological dataset of snakebite patients was analysed to portray snakebite occurrence probability in Chattogram Division, which comprises the south-eastern part of Bangladesh. Five species distribution models were used to map snakebite-prone areas. We found that the study area has large differences in the predicted incidence of snakebite. Our predictions indicate that around 200,000 snakebite cases per year might occur in the study area, where the latest estimate on 2009 estimated less than half that number for this division. Further, predictions for the year 2070 provide us with similar numbers, independent of the global increase in temperature. As snakebite will remain a major health problem for this country in the next 50 years, we propose the development of a sustainable management plan aiming to limit human-snake conflicts and their consequences while safeguarding also snakes and the ecosystem services they provide especially to agriculture.

## Introduction

A better ecological understanding of snakebite is a primary demand of scientists and policymakers. Globally 4.5–5.4 million cases of snakebite per year have been estimated to occur using data from medical records and community surveys [1–4]. As many as 2.7 million of these cases are envenoming bites resulting in approximately 125,000–138,000 human fatalities, 400,000 cases of amputation, as well as other permanent physical and psychological disabilities [2,5,6]. This massive impact on public health demands global coordination [7]. In 2017, the World Health Organization (WHO) put a spotlight on snakebite envenoming and placed it into the priority category ‘A’ of neglected tropical diseases (NTD) [8]. This poverty-related tropical disease is a substantial daily health risk of the rural people in most subtropical and tropical regions [4,9–11], and especially so in rural South Asia [12,13]. Earlier estimates suggest around 34,000 human snakebite fatalities per year in South Asia [2]; however, a much worse scenario is revealed when looking at the individual countries in this region. For instance, more than 50,000 people are dying from snakebite every year in India alone [14–16], more than 8,000 in Pakistan [17], and more than 6,000 in Bangladesh [11], whereas in Sri Lanka annual snakebite fatalities number around 400 [18]. Overall, the death toll from snakebite in South Asia might reach ~75,000, which is almost double the mortality in southeast Asia and Sub-Saharan Africa [13].

Despite the huge death toll, obtaining the actual number of snakebite cases has become one of the main challenges in many snakebite-prone areas. Under-reporting is likely to occur in most African and Asian countries where snakebite is prevalent [1,4,19]. With the notable exception of some countries like Brazil, snakebite is yet to be declared a notifiable disease in most countries including Bangladesh (20). As there is no other recording system available in most developing countries, the hospital-based records provide demographic (age, sex, occupation of the patient etc.), clinical (symptoms of envenoming, administration of antivenom and other medicine, etc.), and epidemiological data (location of the bite occurrence, seasonal fluctuation, types of bite, etc.). This is why the dynamics of snakebite have traditionally been studied using hospital-based data [2,21,22]. Researchers in several countries, however, also have used community-based surveys [11,15,18,23] which revealed that the situation was much worse than previously thought. Considering the latest projected scenario for snakebite, a person is dying every five minutes from snakebite envenoming in any corner of the world [2]. To overcome the limitation of official record systems, the climatic and environmental analysis of snakebite occurrence might provide an alternative way to assess the potential burden of snakebite.

The incidence of snakebite dependents on the distribution and occurrence of snakes, which again is regulated by climatic and environmental variables [24,25]. Studies have shown that climatic events and weather fluctuations are influential in snakebite incidence in the tropics [26]. Particularly, temperature [20], the amount of precipitation [11,27], humidity [28], and also seasonal patterns of precipitation have a significant and predictive effect on snakebite [29]. Studies also suggested that habitat parameters such as land cover, vegetation index, and elevation are associated with the occurrence of snakebite in tropical countries like Sri Lanka [18] and Brazil [20]. Therefore, a combination of climatic, environmental, and human demographic variables may predict the occurrence of snakebite more precisely than traditional methods relying on data gathered from health facilities [24,30].

Being situated where the Indo-Himalayan and Indo-Chinese biodiversity hotspots meet, Bangladesh is home to around 103 snake species, 33 of them being venomous and 13 potentially lethal to humans [31–34]. Among the latter, Elapidae is the largest family of venomous snake in Bangladesh where it is represented by two species of cobra (*Naja kaouthia* and *Naja naja*), one species of king cobra (*Ophiophagus hannah*) and five species of krait (*Bungarus caeruleus*, *Bungarus fasciatus*, *Bungarus lividus*, *Bungarus niger*, and *Bungarus walli*), most of which are found in different parts of the country [33–35]. The family Viperidae is represented by only one species of the true vipers (subfamily: Viperinae), Russell’s viper (*Daboia russelii*), which is restricted to the western part of Bangladesh, and several species of pit vipers (subfamily: Crotalinae), namely green pit vipers (*Trimeresurus* spp.), which are widespread from the central plains to the hills of south-eastern Bangladesh [35]. The venom composition of elapids and viperids are different [36] and the symptom of respective envenoming bites are distinct in human victims [37,38]. Following the development of symptoms and their severity, physicians can predict the snake group responsible for envenomation and decide about the administration and dosage of related medication. Administration of life-saving polyvalent or monovalent antivenom are directly guided by symptoms [39].

Snakebite in Bangladesh has never been studied from an ecological perspective. Earlier studies were focused mostly on epidemiological analyses [11,40,41], clinical manifestations following the bites by different species and biological properties of their venoms [37,38,42], and socioeconomic loss caused by snakebite [43,44]. These studies showed that the study duration was significantly correlated with the number of admitted patients (r = 0.993, p<0.001) (Table 1). Moreover, a wide variation in the results was observed from different studies, such as male/female ratio and age distribution among the bite victims, the ratio of envenoming and non-envenoming bites, etc. (Table 1), and these differences might be due to the short durations of the respective studies. This implies that greater accuracy could be achieved by using a big dataset and also by a systematic recording of all cases preferably from the community level. In addition, such hospital-based dataset can also provide information on responsible voucher specimens and the address of the occurrence location.

**Table 1:** The demography and epidemiology of snakebite in published literature from Bangladesh.

Publication year	Study duration (months)	Total number of patients (n)	Non-envenoming bites (%)	Envenoming bites (%)	Recovery from envenoming bite (%)	Outdoor bite (%)	Indoor bite (%)	Male (%)	Female (%)	Mean age of the patient (yrs.)	Reference
1995	12	44	61	39	94	79	21	68	32	29.6	[45]
1996	32	179	73	27		79	21	66	34	24.8	[46]
1997	13	211	78	22	87	70	30	68.2	31.8	23.8	[47]
1999	12	68	60	40	74	88	12	72	28	26.7	[48]
2008	24	537	68	32				54.2	45.8	27.5	[49]
2010	42	884	60	40		72	28	68	32	26.4	[37]
2011	8	111	90	10	49	53	47	62	38		[50]
2012	30				53.6	86	14	71.4	28.6	31.7	[51]
2012	5	83	54	46	87			71	29		[44]
2013	11	161	55	45	100	100	0	100	0		[52]
2014	24	537	68	32							[53]
2014	18	50	76	24	83	54	46	82	18	38.5	[54]
2015	15	50	40	60	70	55	45	70	30		[43]

The present study aims to contribute to the understanding of the pattern of snakebite occurrence by analysing a large hospital-based dataset from the Chattogram Division of Bangladesh, covering the years 1993 to 2016. Moreover, as species distribution models can be used to map the suitable areas for different snakes, which in turn can be used to infer potential risk areas for snakebite [55,56], we also analysed the influence of climatic variables, forest types, land use types, altitude, and human population density relative to the occurrence of snakebite cases in south-eastern Bangladesh. Integrating such information in predictions on future snakebite occurrences may help to more wisely allocate resources (e.g., antivenom doses) to reduce the impact of this most neglected tropical disease.

## Methods

### Ethics statement

Ethical clearance (Memo no.: CMC/PG/2020/113, Date: 08.10.2020) was obtained from the Ethical Review Committee of Chittagong Medical College (CMC), Chattogram, Bangladesh. We included all the snakebite victims admitted to medical wards, emergency and intensive care units, and excluded the cases from the analysis where the nature of the injury was uncertain (e.g., other animal bite or poisoning events) that might confuse the clinical profile of snakebite.

Information about patients admitted with a history of snakebite has been recorded at Chittagong Medical College Hospital (CMCH) in Chattogram, Chattogram Division, Bangladesh, since 1993 through a dedicated 24/7 ‘snakebite clinic’. Because there were no other dedicated snakebite clinics available, patients from any terminal point in the medical network from the entire division are referred to this clinic to receive modern medical treatment. As the recording process was interrupted for some years, data from eight years (2004–2011) were excluded from analysis. Here, we compiled only a 16-year record (1993–2003 and 2012–2016) from the total data for the present study. Verbal consent for using patient data for research only was taken from the patient or his/her next of kin in case patients were unable to communicate, and confidentiality of the information was maintained. Our co-authors AG, RA, UK, and MAF are actively contributing to the establishment and maintaining this record register.

### Sub-dataset selection

We compiled a primary database of 4371 cases of snakebite in a single-point record at CMCH, but this database has absence of information in many entities or those were accidentally left empty while recording in the original document or patient referred from outside the Chattogram division. Due to those circumstances, a fair amount of information was discarded and sub-datasets were extracted from this primary database. One sub-dataset consisting of 3972 cases contained complete information about the gender of the snakebite victim was used for demographic analysis. Similarly, a set of 4268 cases with address up to subdistrict level within this division was used to produce district and sub-district maps of snakebite incidence, while another set of 3958 cases with further address of the bite occurrence was used to extract geographical coordinates. A total of 4331 cases that included information on the date of snakebite occurrence was used to study the seasonal influence on snakebite variation. Snakebite cases in each of the three seasons was calculated by averaging the number of cases in that season over the study period. A total of five variables, i.e., age and gender of the patient, bite date and geographical position of the snakebite incident, and bite type were selected for further analysis. The fifth variable “bite type” was determined by the respective physician depending on the snake specimen brought with the patient and/or the bite marks and/or symptoms of envenoming [39,57,58]. This parameter was determined by co-author AG, RA, UK, MAF at different times supervising the physician in diagnosis of the bite type at CMCH. In total five categories of snakebites were determined, i.e., cobra bite, green pit viper bite, krait bite, non-envenoming snakebite, and other envenoming snakebite.

### Study area

The study area, Chattogram Division, is the largest division of Bangladesh. It is located in the southeast of the country and consists of eleven districts. The area of Chattogram Division is 34,529.97 km^2^ in size and is dominated by a maritime climate and tropical hilly vegetation. The area generally experiences three distinct seasons: the hot summer, which extends from March to June, followed by the Monsoon or Rainy season from July to October, and finally, the Winter season, which lasts from November to February. Its human population density is ~850 persons per square kilometre. The districts in the western plains of this division have a dense human population, whereas the hilly districts with tropical forests in the eastern part are the least populated [59]. This division contains around 20% (human population is 31,176,438 in 2020) of the total population of the country, and human density in this division is increasing by ~6% every year [59]. Biodiversity is similar across the study area and consists of hilly forests and floodplain ecosystems where about 60 snake species are known to occur [35].

### Predictors

Nineteen bioclim variables in 1x1 km^2^ resolution for current and future (the year 2070) climate were collected from worldclim2.0 (http://www.worldclim.org, accessed on 14.06.2019), and Community Climate System Model in RCP6.0 projection was used in the future projection of snakebite incidence for the year 2070 [60,61]. The land use/land cover (hereafter denoted as ‘landuse’) raster was in 100x100 m^2^ pixel resolution, and the updated forest cover (forest) raster was in 30x30 m^2^ pixel resolution; both were collected from the Copernicus global land service [62,63]. The SRTM 3 arc-second (90x90 m^2^) land surface elevation (elevation) data were extracted from the Earth Resources Observation and Science Centre [64]. The nearest neighbour resample technique in ArcGIS was used to resample these three raster files to 1x1 km^2^ of spatial resolution. The global total population projection raster file based on the Shared Socioeconomic Pathways (SSPs) at a resolution of 1x1 km^2^ was downloaded from the Socioeconomic Data and Applications Center (SEDAC, https://sedac.ciesin.columbia.edu, accessed on 19.10.2019) [65]. Shared Socioeconomic Pathways describe alternative future trends of societal factors (such as demographics, economics, technological development, governance, etc.) that can be combined with climate projections to carry out integrated analyses [66]. To reduce bias of the predictors, the values of raster layers were standardized by the *scale()* function in the raster stack [67]. In the case of a Pearson correlation coefficient >0.9 between any two rasters, the one with less interest was omitted. This will reduce the multicollinearity in further analyses. For instance, the precipitation of wettest quarter (Bio16) and precipitation in wettest month (Bio13) is highly correlated, here the precipitation of wettest quarter was selected as better presentation of precipitation in the whole rainy season over a month of the rainy season. Finally 18 predictor raster files were selected and cropped by the spatial polygons of the country obtained from the open licence repository [68]. All raster files were in the WGS84 projection. R platform was used to process all GIS layers, using raster [67], rgdal [69], maptools [70], sp [71], and sf [72] packages.

### Model analysis

After scrutiny and removal of entries with inadequate geo-reference information, a total of 998 snakebite occurrence points were selected from the hospital records and 1,000 background absence points were randomly generated from study area polygon using the *randomPoints()* function [73]. These two sets of coordinates were combined to make a presence-absence geolocation list of snakebite. Values of the independent variables were extracted from the raster stack of predictors using these coordinates. This database was used to identify the responsible predictors of snakebite occurrence and snakebite prone areas by using principal component analysis (PCA) and ecological niche models, respectively. Eighty percent of this presence-absence dataset was used to train the model, and the remaining 20% was used to test it. We used a climate envelope model (Bioclim), two regression models (Generalized Linear Models using Binary and Gaussian probability distribution), and two machine learning models (Maxent and Random Forest) in their default options. The Bioclim algorithm evaluates location similarity by comparing environmental variables at a given location with a percentile distribution derived from known occurrence locations. In the case of the generalized linear model, the response variable indicates the absence or presence of snakebite occurrences within each pixel of the study area in Binary distribution, while the Gaussian distribution represents the total count of snakebites within a grid cell. Maxent, an abbreviation for maximum entropy modelling, predicts species occurrences by identifying the distribution that is most widely dispersed or approaches uniformity while considering constraints imposed by environmental variables at known locations. On the other hand, Random Forest utilizes data subsets and features to construct a forest of decision trees, which helps mitigate model overfitting by averaging these trees and demonstrates reduced sensitivity to data noise and outliers. It is worth noting that each model may have its own algorithmic limitations in prediction, but these limitations can be minimized by combining outcomes of the different algorithms. The *predict()* [67] function was used to predict the probability of snakebite occurrence for each square kilometre area (equal to cell size in each raster file) within the study area for both the present and the year 2070. Consensus maps were generated by using a *weighted mean()* function to combine predicted maps from five models [74]. The *evaluate()* [73] function was used to determine AUC, correlation coefficient, and p-value to evaluate model success. These analyses were conducted using the ‘sdm’ [74], ‘raster’ [67], and ‘dismo’ [73] packages in the R platform. A logistic regression equation was used to predict the number of snakebite cases through determining the effect size (B value) on the response variable. Here, backward method was used to exclude the insignificant or the least significant variables from each itinerary and the highest cumulative effect (adjusted R^2^) with the least number of predictors was considered in the estimation snakebite occurrence. The significance level (p-value) was expressed as * is p<0.05, ** is p<0.01, and *** is p<0.001 from this point forward.

## Results

**Fig 1:**
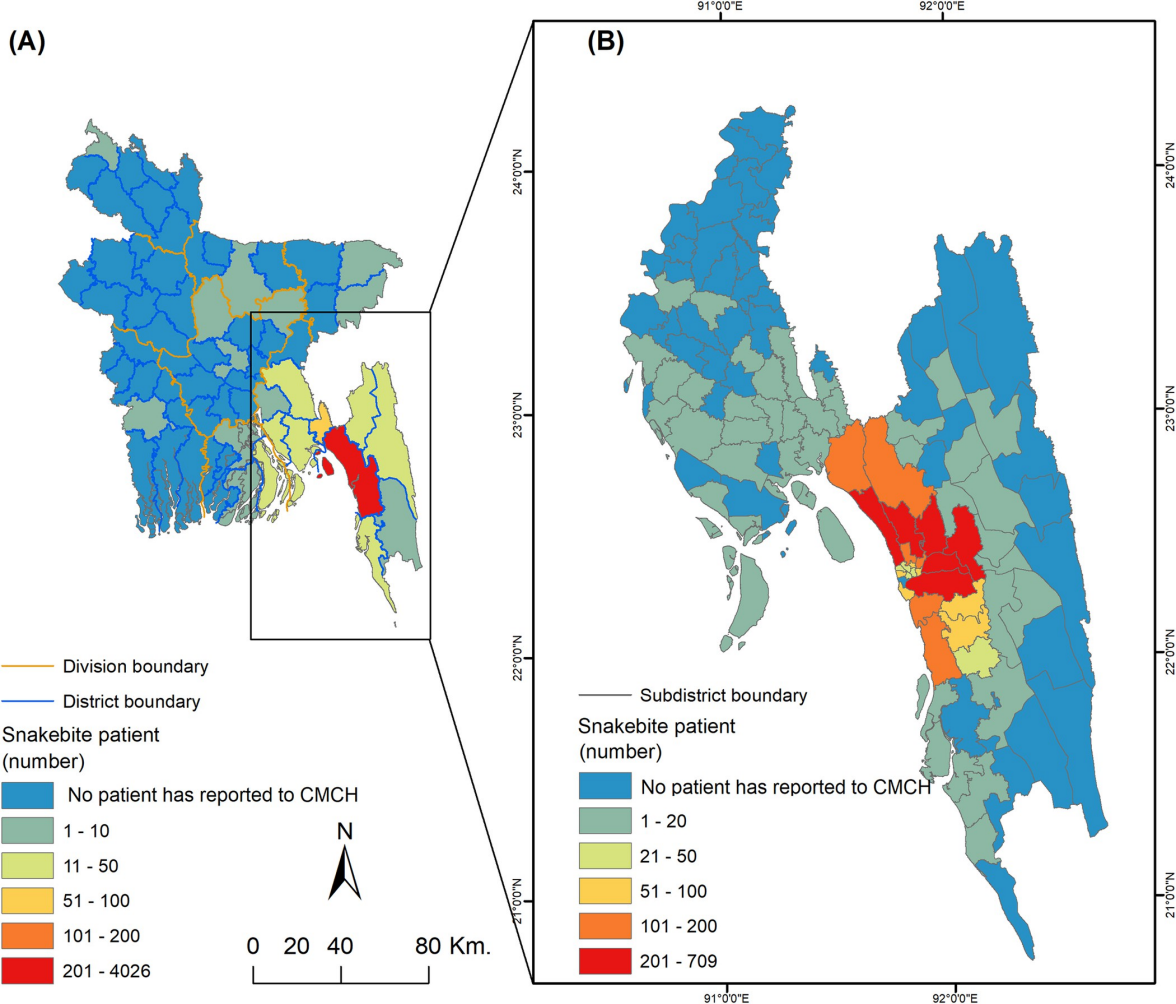
(A) District- and (B) sub-district-wise distribution of snakebite cases recorded in Chittagong Medical College Hospital from 1993–2016. The source country map was collected from https://www.diva-gis.org/gdata. This GIS file is free to research use (https://www.diva-gis.org/node).

Among the patients with snakebite incidents that were admitted to Chittagong Medical College Hospital (CMCH), 99% lived in the study area, Chattogram Division, Bangladesh (Fig 1).

### Demographics and epidemiology

**Fig 2:**
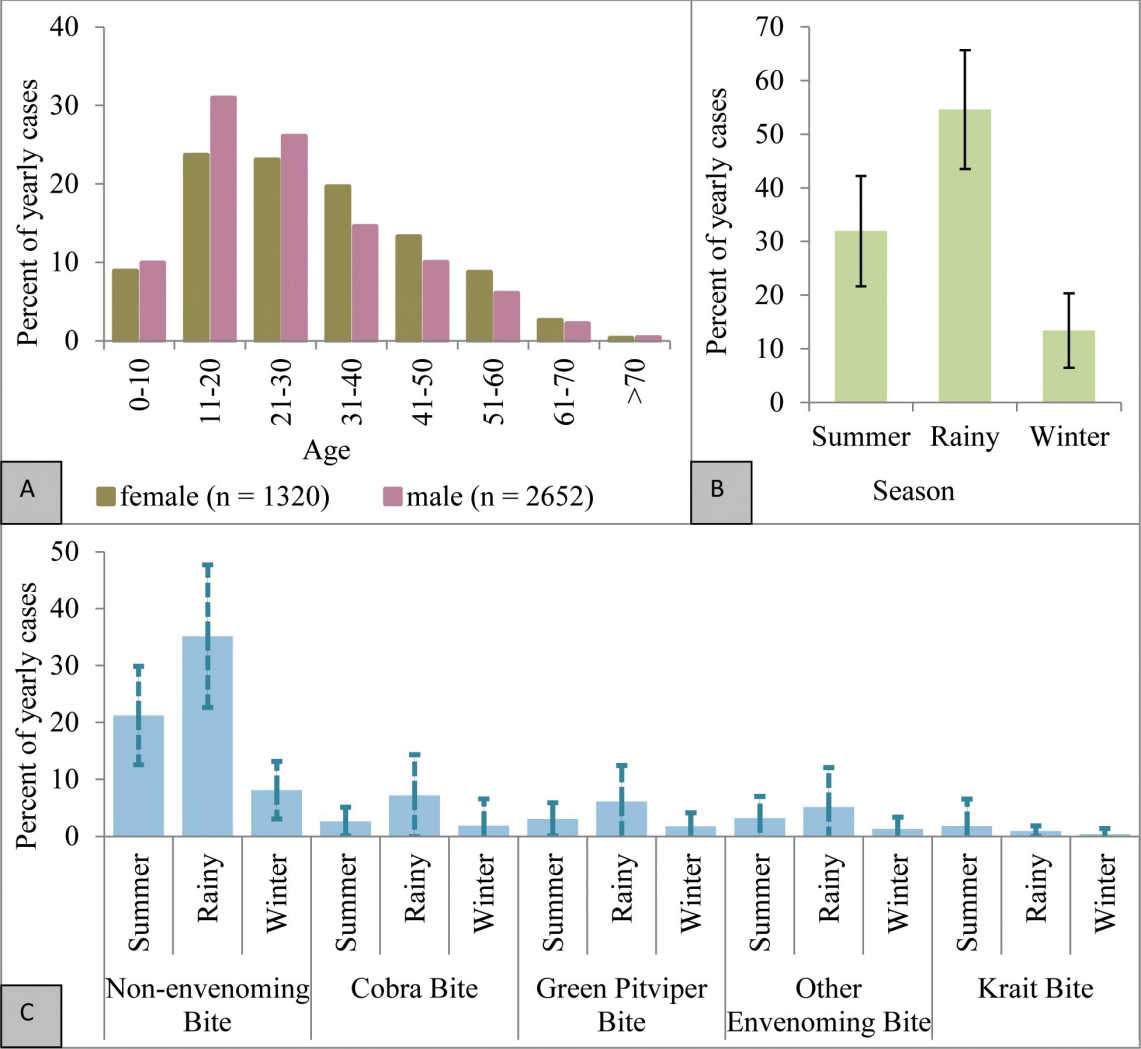
(A) Sex ratio of snakebite victims across different age groups, (B) seasonal distribution of total bite cases, and (C) distribution of five clinically identified snakebite categories during the three major seasons of Bangladesh.

About 67% (2652 patients) of the snakebite patients were male and 33% (1320 patients) were female. The number of male patients was higher than females in every age group (Fig 2A). Around 70% of patients belonged to the 10–40 year age group. Children below 10 years were as vulnerable as teenagers whereas elderly people were the least affected. The sum of “non-envenoming snakebite” and “other envenoming snakebite” was always higher than that of envenoming snakebites attributed to one of the genus-level categories. The latter consisted of around 35% of the yearly snakebites (Fig 2C). Of the four categories of envenoming snakebites, cobra bites were in the top rank constituting on average ~12% of the yearly recorded envenoming snakebite cases, followed by green pit viper bites (~11%), and “other envenoming snakebites” (~10%) (Fig 2C).

### Seasonal variation in snakebite occurrence

Of the three major seasons in Bangladesh, the rainy season (from July to October) is the one with the highest number of snakebites, including almost 55% of the total snakebite cases per year, followed by the summer season, extended from March to June (Fig 2B). A similar trend is also reflected in the seasonal distribution of the various types of snakebite (Fig 2C). In the summer, the green pit vipers (*Trimeresurus* spp.) together with “other venomous snakes” contributed to the major portion of envenoming bites in the study area. During winter, however, cobra bites and green pit viper bites together constituted 13% of the total annual number of snakebites (Fig 2C). Krait bites were the least common throughout the year.

### Influential variables

Of the 18 predictors, 65.6% of the total variance was described by two principal components (PCs). The analysis revealed that PC1, a combination of precipitation parameters and elevation, described 33.5% of the total variance of the variables. A combination of temperature parameters with elevation, PC2, described 32.1% of the variance. Backward regression analysis revealed that the cumulative impact of ten significant predictors of snakebite occurrence was 76% (p<0.001) (Eq 1).


$$R^{2}=\;13.299\;(Annual\;Mean\;Temperature)*+5.196\;(Mean\;Diurnal\;Range)*–5.075\;(Isothermality)***–5.338\;(Mean\;Temperature\;of\;Wettest\;Quarter)*–3.61\;(Annual\;Precipitation)***+0.275\;(Precipitation\;\mathrm{of}\;\mathrm{Driest}\;\mathrm{Month})*+5.068\;(\mathrm{Precipitation}\;\mathrm{Seasonality})***+0.309\;(\mathrm{Precipitation}\;\mathrm{of}\;\mathrm{Warmest}\;\mathrm{Quarter})*+0.601\;(Elevation)*+1.573\;(Human\;Population)***–0.778.$$ Eq 1

In this analysis, temperature-derived variables were shown to have a bi-directional influence on snakebite occurrence. Individually, annual mean temperature and mean diurnal fluctuation have a positive effect on snakebite occurrence, while isothermality and high temperature in the rainy season have a negative impact. Except for annual rainfall, all precipitation variables are positively influential on snakebite. The impact of different forest and land use types was found to be insignificant, while other variables such as human population and elevation were positively related to but with a low impact on snakebite occurrences.

### Prediction of snakebite occurrence

**Fig 3:**
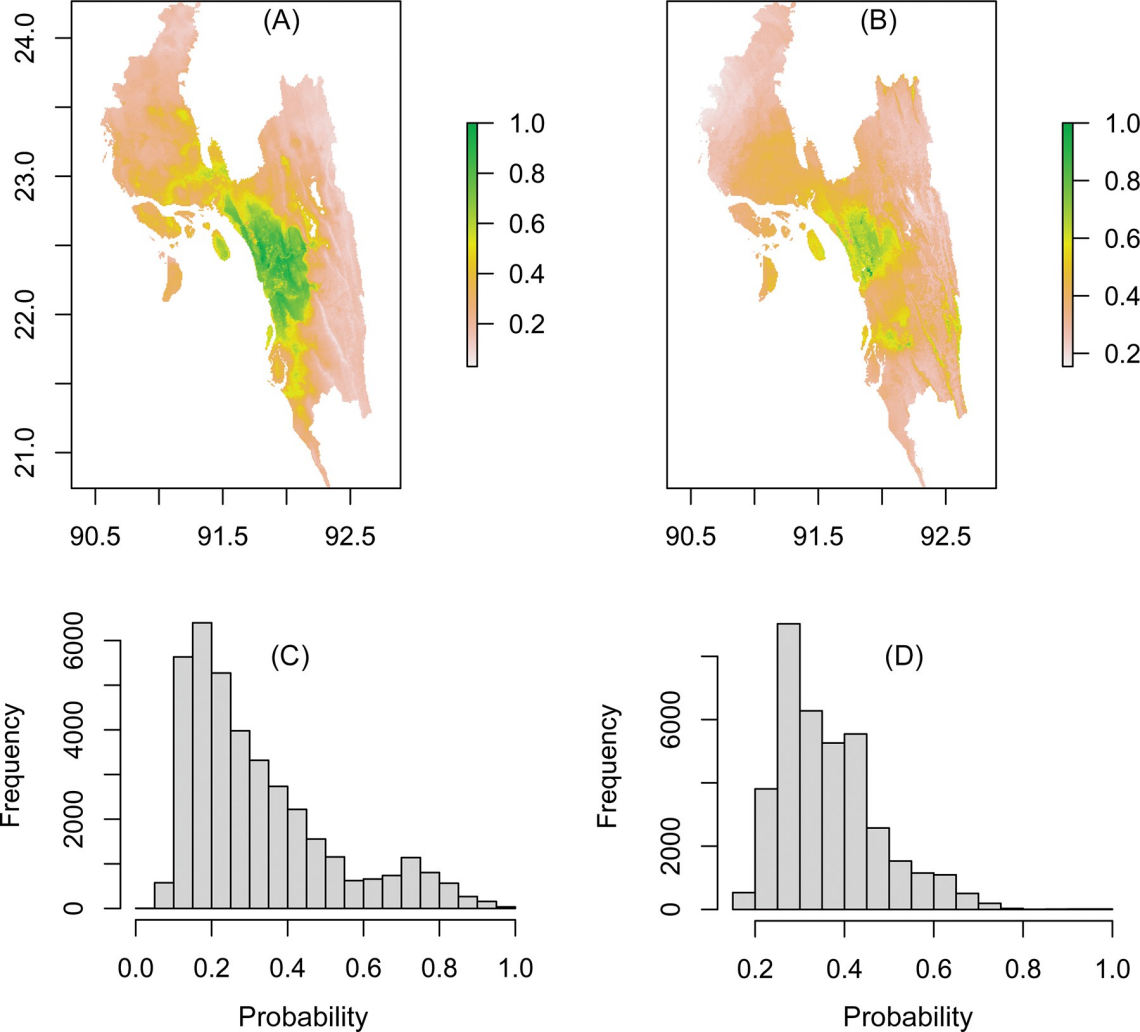
(A) Current and (B) future consensus prediction map of snakebite occurrences in Chattogram Division as produced by the weighted mean of prediction by five different models. The scale presents the probability value. Below: Histogram of the cell frequencies with various probability values in (C) current time and (D) in the year 2070. The source country map was collected from https://www.diva-gis.org/gdata. This GIS file is free to research use (https://www.diva-gis.org/node).

**Fig 4:**
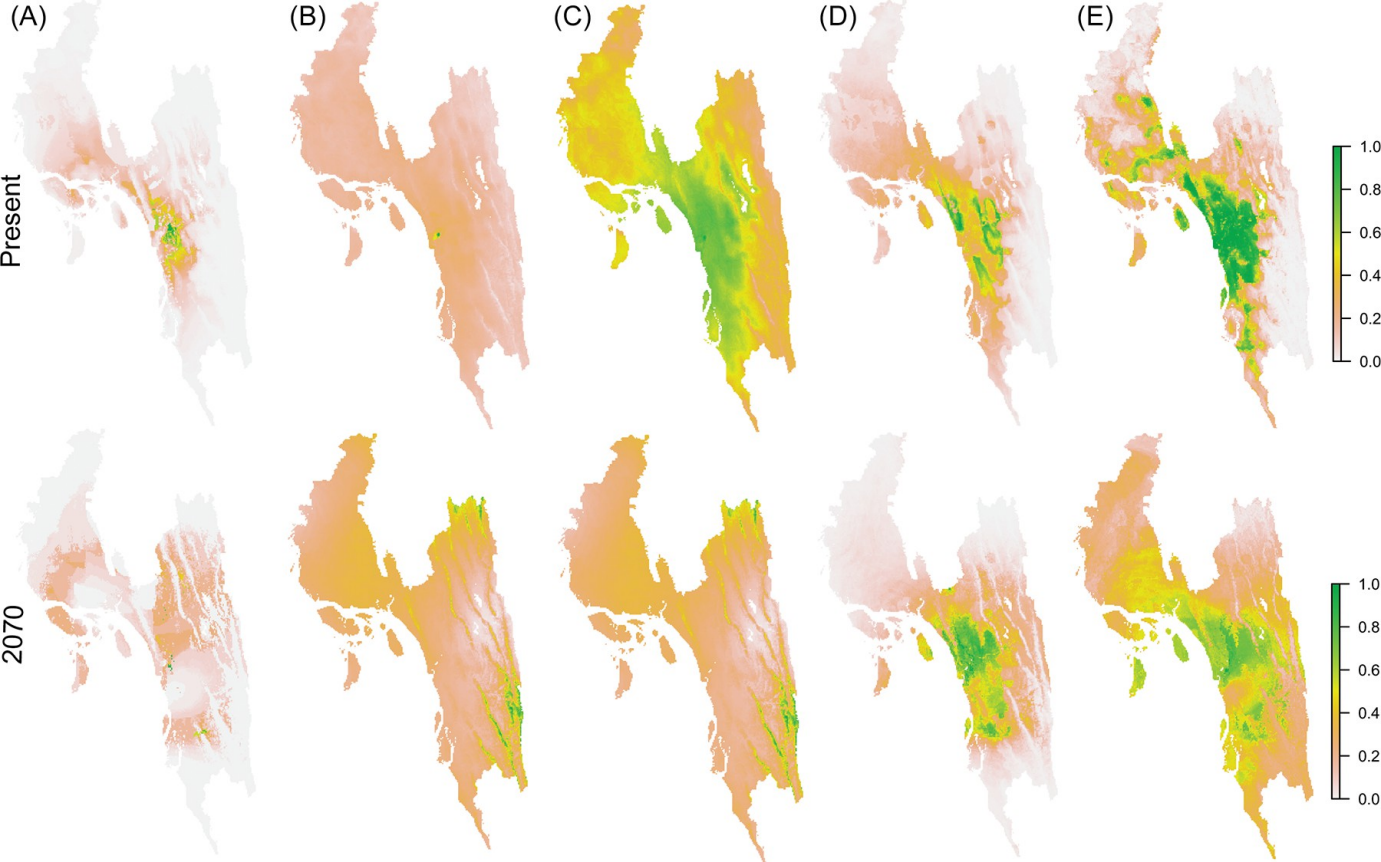
The map was generated by five different models to show snakebite occurrence in Chattogram Division. (A) Bioclim model, (B) Generalized Linear Model (Binomial), (C) Generalized Linear Model (Gaussian), (D) Maximum Entropy (Maxent) Model, (E) Random Forest Model. The first row represents the prediction for the current time; the second row represents the prediction for the year 2070. The source country map was collected from https://www.diva-gis.org/gdata. This GIS file is free to research use (https://www.diva-gis.org/node).

**Fig 5:**
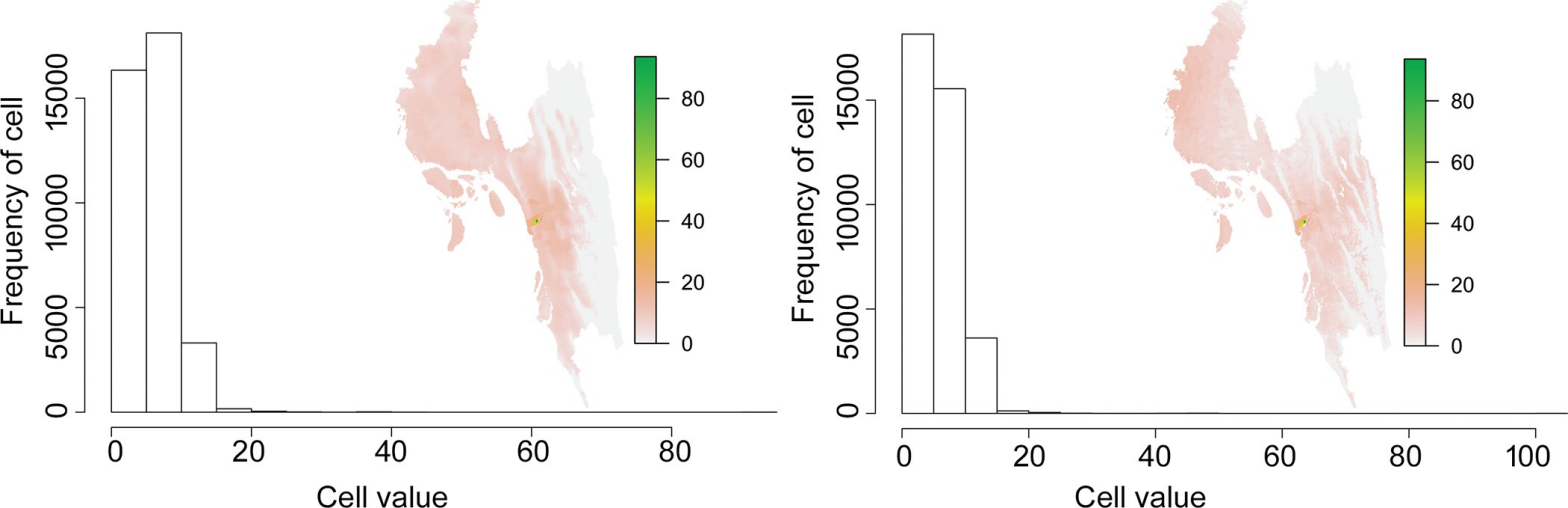
Estimation of snakebite incidence for (A) present time and (B) the year 2070 using Eq 1. Colour bars represent human density per square kilometre (in every cell of the raster file) at risk of snakebite. The source country map was collected from https://www.diva-gis.org/gdata. This GIS file is free to research use (https://www.diva-gis.org/node).

Apparently, the prediction maps identified the hilly areas in the eastern part of the study area, to have a lower the probability of snakebite (Figs 3 and 4). Although every model showed a high success (~90%) rate of prediction (Table 2), the sizes of the snakebite prevalence area predicted by the models are slightly different (Fig 4). Among the models used, the one with the smallest predicted snakebite prevalence area was Bioclim, while Generalized Linear Models (GLM) predicted that snakebite may occur in the entire study area (Fig 4). The future consensus maps show that snakebite may occur throughout the study area with a slight decrease in numbers (Figs 3 and 5). Compared to the prediction for present-day conditions, snakebite risk will increase in the eastern part of the study area in the near future (Fig 3B). As per Eq 1, more than 200,000 snakebite episodes might occur in Chattogram Division every year. These numbers remain almost the same (191,612 incidents) in the case of the prediction for the year 2070 (Fig 5).

**Table 2:** Results of the five models used in the training and prediction of snakebite occurrences.

Model	AUC	Correlation coefficient, p-value
Bioclim	0.890	0.492, p < 0.000
GLM (function Binomial)	0.940	0.603, p < 0.000
GLM (function Gaussian)	0.938	0.775, p < 0.000
Maxent	0.914	0.684, p < 0.000
Random Forest	0.937	0.811, p < 0.000

## Discussion

The results of this study show that snakebite may occur in the entire study area in Chattogram Division, Bangladesh, but snakebite risk varies across the map. Snakebite risk is predicted to be high in flood plains in the western part of the study area, whereas the prevalence is low in the hilly eastern side. This indicates that the positive relation between elevation and snakebite (Eq 1) is non-linear and may not positive in case of high elevation. For instance, elevated places in floodplain provide shelter to snake but hilly terrains (600–900 metres high) in the eastern part of the study area limit the distribution of the snake which may decrease the snakebite possibilities. This also might be due to differences in human population density as the plains are more populated than the hilly terrain [59]. In addition, the probability of snakebite occurrence is high in areas close to the city border. A higher density of the human population lives in suburbs than in the countryside [59], and this might cause a higher snakebite occurrence in these areas. Also, health awareness and facilities are comparatively more up-to-date in the city and its adjacent areas than in the village areas. Hence, snakebite patients from the suburbs have a higher chance to report to the hospital and to get into the clinical database we have maintained and analyzed. According to these records, there is an absence of snakebite cases from some sub-districts that are located far from the city (Fig 1), however, models have corrected those absence values and suggested that snakebite may occur in those areas, too (Figs 1 and 4). This supports the view that the true snakebite incidence could be much higher than that reported to hospitals [75]. Moreover, insufficient medical assistance in rural areas causes a higher proportion of snakebite fatalities, which remains out of official records [9,42]. Unreliability of the current recording and reporting system used by health care facilities demand that snakebite is legislated to be a notifiable disease.

Previously, a postal survey conducted in the year 1995 had suggested that 4.3–7 snakebite cases per 100,000 people per year might occur in Chattogram Division [40,41], which would correspond to 2,000 snakebite episodes per year among the 28.42 million population of this division during the study period. In the year 2010, Rahman et al. [11] estimated a snakebite incidence of 397.8 (211.8–680.3, 95% CI) per 100,000 population-year in this division after conducting a nationwide community-based cluster survey in rural areas of Bangladesh. This would correspond to around 100,000 snakebite cases annually in our study area. Based on the present analysis, our estimation of the number of people vulnerable to snakebite suggests an even worse scenario, doubling (more than 200,000 incidents) the number obtained by that latest community-based study [11]. A variety of reasons have already been identified that cause a large portion of snakebite victims worldwide to remain invisible to official records and studies [19]; our estimation using climatic, environmental, and human demographic variables through several robust statistical models might improve the understanding of the true scenario. Interestingly, our projections of future scenarios also suggest that the snakebite incidence in Chattogram Division might remain almost the same (191,612 incidents) under the changing climate and human population density of the year 2070, when the predicted human population of this division will be more than 31.5 million [65].

According to the predicted climate change (RCP 6.0) scenario, the incidence of snakebite might increase in moderate altitudes in hilly habitats of Chattogram Division in the next 50 years (Figs 3B and 4), supporting the suggestion that the spatial distribution of snakebite might be altered due to global climate change [24], with an increasing trend in the future [76]. Higher altitudes may in the future provide cooler shelter from too much heat in the lowlands to medically highly important snake species [77], which may increase snakebite incidence, fatalities and disabilities in hilly areas. At the same time, venomous snake species that are adapted to high-altitude temperate climates, and are usually medically less important, may become rare or extinct due to global warming [78], giving way to upward dispersal by more dangerous lowland species. Due to its association with climate change, snakebite envenoming must therefore be considered and designated a climate-sensitive disease. A snakebite management plan can use these predictions to increase its sustainability.

Global environmental changes including the modification of habitats, ecosystems, forest structures, and types of land use may greatly impact the suitability of reptile habitats [79–82]. To obtain a successful snakebite management plan, climate change and other environmental changes should therefore be considered in health policy-making. Moreover, the projected increase of the human population in Bangladesh up to 260 million in the year 2100 [83] has the potential to increase snake-human encounters and conflicts. Thus, taking into account the synergistic influence of different global and regional changes, and hence adding more variables in analyses, is likely to improve the accuracy and utility of estimations.

Around 60 terrestrial snake species, including the highly venomous cobras (*N*. *kaouthia*, *N*. *naja*), king cobra (*O*. *hannah*) and kraits (*B*. *caeruleus*, *B*. *fasciatus*, *B*. *lividus*, *B*. *niger*, *B*. *walli*) occur in the study area [35,84]. In the present study, a clear seasonal influence, with a peak of snakebite during the monsoon season, could be recorded (Fig 2) in agreement with earlier studies from this area [37]. A low frequency of snakebite was observed in the cool and dry winter season, with increases after precipitation [29]. Moreover, high humidity in the study area increased the risk of snakebite, as observed previously in other tropical areas [28]. Particularly during monsoon, i.e., from July to September, venomous snakes are very active, and this high snake activity coincides with a peak period for agricultural work [11,37]. The study area is classified as belonging to the south-eastern climatic subzone [85,86], with temperatures fluctuating between 32°C and 13°C, and annual precipitation being usually over 2,540 mm, with heavy rainfall in the monsoon season. As a consequence, floods are a regular natural calamity in Bangladesh and snakes living in floodplain ecosystems like the north-western part of the study area face temporarily unsuitable habitat conditions during flood events [87]. At such times, snakes usually take shelter on higher land or inside human habitations, which increases the conflict between humans and snakes during floods [88]. This also supported by our analysis, where snakebite in positively correlated to elevated places in flood plains but not hilly elevation. This demands further attention to minimize the fatalities of envenoming snakebite in flooded area. Furthermore, the occurrence of the two species of cobra, several kraits, and vipers in village forests and inside human habitations is common in Bangladesh [35]. The forest cover in the study area mostly includes two formations, i.e., hilly forest referring to evergreen trees and dense undergrowth on elevated land, and village forest referring to patches of forest surrounding rural households and in barren areas dominated by shrubs, herbs, and undergrowth. Similarly, the study area is dominated by either a hilly ecosystem or a floodplain ecosystem [89]. As these forest and ecosystem types are widespread in the study area, their distribution was not found to be influential on snakebite occurrence in this division of Bangladesh.

Our analysis of a large clinical 16 year-dataset revealed that the average proportion of non-envenoming bites among patients presenting to our hospital was 65% (the lowest was 20% in 1993 and the highest 85% in 2009) which appeared to be fine-tuned comparing the previous studies. An earlier study reported that around 60% of snakebite patients admitted to CMCH between 1999 to 2002 had non-envenoming bites [37]. In other areas of Bangladesh, around 66% of snakebite victims have been reported as having non-envenoming bites (Table 1). Among envenoming bites, cobra bites were the main cause of human envenoming in the study area. So far, all cobra bites treated at CMCH that could be diagnosed to species level were caused by *N*. *kaouthia* [38]. However, krait bites, while making up only 3% of annual hospitalized snakebite cases in our study area, have a higher fatality rate due to the absence of appropriate antivenom, higher likelihood of complications, and limited facilities for providing assisted respiration which is required more often and for longer periods in patients with neurotoxic envenoming following krait bites. In addition, envenoming by *B*. *niger* in the study area has been shown to cause generalized rhabdomyolysis and acute kidney failure in addition to neuromuscular paralysis, resulting in the need for providing limitedly available dialysis in addition to assisted ventilation [42]. In earlier years and elsewhere in Bangladesh the fatality rate of snakebite cases reported to hospitals was indicated as being around 20–22% [41,47,48,90,91] (Table 1), but it is currently much lower in CMCH (<1%) which highlights the value of trained healthcare staff, availability of antivenom, and protocol-based clinical management of snakebite patients in a dedicated ward. Our analysis suggests that males may be three times more vulnerable to snakebite than females in this region of Bangladesh, and that the younger age groups are more frequently affected than senior citizens. In previous studies, the number of male victims was reported to be five times higher than that of females (Table 1), which is a notable difference when compared to the results of our analysis of the big dataset from the Chattogram Division. This may reflect regional differences in the pursuit of high-risk activities, but it is also possible that this male/female bias is influenced by differences in awareness and treatment-seeking behaviour, which might differ between regions or could have changed over time. For example, CMCH physicians have carried out numerous awareness campaigns and outreach activities in the rural catchment area of their hospital over the last 25 years. Such activities are mostly missing in the rest of the country, but they would be expected to have contributed to changes in treatment-seeking behaviour in parts of the study area. Regarding actual exposition to contact with snakes, men in Bangladesh are more often involved in outdoor professions such as fishing, collection of forest materials, and work in agriculture than women, so they have a higher chance of encountering snakes in the wilderness [9,11]. The present study found that the most vulnerable human age group in Chattogram Division was the one between 20 and 40 years of age, and the proportion of envenoming snakebite in children (0–10 years) was ~10% of the total (n = 2772), much higher than that of older age groups (>60 years). These findings confirm previous observations indicating that people of young age groups and those engaged in outdoor activities are at higher risk of snakebite [11,44].

Although most snakebite incidents happen outside human housing, 12–47% of the annually reported snakebites in north-western Bangladesh occurred inside houses [50]. As various species of krait (genus *Bungarus*) are especially notorious for living inside or entering houses where they inflict bites on sleeping people [92,93], and because krait bite envenoming is especially difficult to treat, snakebite at night inside the house is often associated with a particularly high risk of severe neurotoxic envenoming and hence fatal outcome [94]. The blessings of a great snake species diversity and the occurrence of snakebite everywhere have turned this terrifying experience into a daily life event in rural Bangladesh, where it is a major poverty-related disease [45,95]. However, the current economic rise of the country offers perspectives for much-needed improvements in housing conditions, farming practices, and healthcare services, which in turn may lead to less frequent human-snake conflicts resulting in fewer snakebite cases, and better outcomes for those who are bitten.

In snakebite envenoming, the use of antivenom is the only approved specific treatment. However, the neutralizing capacity of antivenom is sensitive to inter- and intraspecific snake venom variation [96,97]. Thus, antivenom produced from evolutionarily distant species often fails to neutralize medically important toxins of those venoms which were not included in the immunizing mixture [97]. Thus, it is important to use venoms from local snake populations in antivenom production to account for intra-specific venom variation [98,99]. The WHO has recently defined the urgent target of reducing human mortality and disability due to snakebite by half by the year 2030 [99]. An integrated global strategy equipped with scientific input and backed with robust statistical analysis has long been demanded by scientists and policymakers [19,100]. Our study suggests that snakebite management, including public awareness programmes with locally adapted strategies to change treatment-seeking behaviour, improve medical support and the training of physicians, should be widely available at the primary healthcare level in Bangladesh to minimize the number of snakebite cases, deaths and disabilities. In addition, the distribution and availability of antivenom and other associated medicines, life-saving logistics, facilities and skills like those associated with the provision of respiratory and renal support, are of key importance to reducing the burden of disease, disability, and death from snakebite in Bangladesh and elsewhere.

## Conclusion

The present study provides a scientific approach to predicting the incidence of snakebite envenoming, a neglected tropical disease, in Bangladesh. Our analyses show that snakebite occurs across the entire study area, with different probability values, and strongly suggest that the real number of snakebite events may be higher than that registered in official health databases. According to our estimation, around 200,000 people per year are bitten by snakes in Chattogram Division, more than twice the previous estimate for this division of Bangladesh. While the hilly and sparsely populated area of the Chattogram Hill Tracts of Bangladesh is presently found less snakebite prone in our analysis, projections of future scenarios suggest that these areas may have an equally high probability of snakebite as the flood plains. Our analyses also allowed us to identify areas with a high probability of snakebite from where no or insufficient official records of snakebite had been forthcoming, pointing towards previously overlooked and sketched areas. Our maps and estimations can therefore be used to enhance the allocation of healthcare services with limited resources. A sustainable snakebite management plan might be improved by using the future predictions: 1) denoting snakebite-prone areas, 2) prioritising public awareness programs in those areas, 3) possible patient size to deal with related to hospital, medicine, and antivenom supply, 4) established a recording and reporting system in health care facilities with snakebite designated as a notifiable health condition. Bangladesh needs to secure safe, effective and affordable antivenoms, supportive drugs and facilities, including trained physician teams, to fight against this highly prevalent disease of poverty.

## Supporting information

S1 Data Anonymized dataset version of the original data derived from hospital records used in this study. (XLSX)
